# STAT5 is Expressed in CD34^+^/CD38^−^ Stem Cells and Serves as a Potential Molecular Target in Ph-Negative Myeloproliferative Neoplasms

**DOI:** 10.3390/cancers12041021

**Published:** 2020-04-21

**Authors:** Emir Hadzijusufovic, Alexandra Keller, Daniela Berger, Georg Greiner, Bettina Wingelhofer, Nadine Witzeneder, Daniel Ivanov, Emmanuel Pecnard, Harini Nivarthi, Florian K. M. Schur, Yüksel Filik, Christoph Kornauth, Heidi A. Neubauer, Leonhard Müllauer, Gary Tin, Jisung Park, Elvin D. de Araujo, Patrick T. Gunning, Gregor Hoermann, Fabrice Gouilleux, Robert Kralovics, Richard Moriggl, Peter Valent

**Affiliations:** 1 Ludwig Boltzmann Institute for Hematology and Oncology, Medical University of Vienna, 1090 Vienna, Austria; daniela.berger@meduniwien.ac.at (D.B.); daniel.ivanov@meduniwien.ac.at (D.I.); yueksel.filik@onc.lbg.ac.at (Y.F.); peter.valent@meduniwien.ac.at (P.V.); 2 Department/Hospital for Companion Animals and Horses, University Hospital for Small Animals, Internal Medicine Small Animals, University of Veterinary Medicine Vienna, 1210 Vienna, Austria; 3 Department of Internal Medicine I, Division of Hematology & Hemostaseology, Medical University of Vienna, 1090 Vienna, Austria; alexandra.keller@meduniwien.ac.at (A.K.); florian.schur@ist.ac.at (F.K.M.S.); christoph.kornauth@meduniwien.ac.at (C.K.); 4 Department of Laboratory Medicine, Medical University of Vienna, 1090 Vienna, Austria; georg.greiner@meduniwien.ac.at (G.G.); nadine.witzeneder@meduniwien.ac.at (N.W.); gregor.hoermann@meduniwien.ac.at (G.H.); 5 Institute of Animal Breeding and Genetics, University of Veterinary Medicine Vienna, 1210 Vienna, Austria; bettina.wingelhofer@manchester.ac.uk (B.W.); heidi.neubauer@vetmeduni.ac.at (H.A.N.); richard.moriggl@vetmeduni.ac.at (R.M.); 6 INSERM, ERI-12, Faculté de Pharmacie, Université de Picardie Jules Verne, 80000 Amiens, France; emmanuel.pecnard@univ-tours.fr (E.P.); fabrice.gouilleux@univ-tours.fr (F.G.); 7 Research Center for Molecular Medicine (CeMM), 1090 Vienna, Austria; harini.nivarthi@meduniwien.ac.at (H.N.); robert.kralovics@meduniwien.ac.at (R.K.); 8 Department of Pathology, Medical University of Vienna, 1090 Vienna, Austria; leonhard.muellauer@meduniwien.ac.at; 9 Department of Chemistry, University of Toronto, Toronto, ON M5S 1A1, Canada; gary.tin@mail.utoronto.ca (G.T.); ji.park@mail.utoronto.ca (J.P.); e.dearaujo@mail.utoronto.ca (E.D.d.A.); patrick.gunning@utoronto.ca (P.T.G.); 10 CNRS UMR 6239, GICC, Faculté de Médecine, Université François Rabelais, 37020 Tours, France

**Keywords:** MPN, STAT5, JAK2 V617F, neoplastic stem cells

## Abstract

Janus kinase 2 (JAK2) and signal transducer and activator of transcription-5 (STAT5) play a key role in the pathogenesis of myeloproliferative neoplasms (MPN). In most patients, *JAK2* V617F or *CALR* mutations are found and lead to activation of various downstream signaling cascades and molecules, including STAT5. We examined the presence and distribution of phosphorylated (p) STAT5 in neoplastic cells in patients with MPN, including polycythemia vera (PV, *n* = 10), essential thrombocythemia (ET, *n* = 15) and primary myelofibrosis (PMF, *n* = 9), and in the *JAK2* V617F-positive cell lines HEL and SET-2. As assessed by immunohistochemistry, MPN cells displayed pSTAT5 in all patients examined. Phosphorylated STAT5 was also detected in putative CD34^+^/CD38^−^ MPN stem cells (MPN-SC) by flow cytometry. Immunostaining experiments and Western blotting demonstrated pSTAT5 expression in both the cytoplasmic and nuclear compartment of MPN cells. Confirming previous studies, we also found that JAK2-targeting drugs counteract the expression of pSTAT5 and growth in HEL and SET-2 cells. Growth-inhibition of MPN cells was also induced by the STAT5-targeting drugs piceatannol, pimozide, AC-3-019 and AC-4-130. Together, we show that CD34^+^/CD38^−^ MPN-SC express pSTAT5 and that pSTAT5 is expressed in the nuclear and cytoplasmic compartment of MPN cells. Whether direct targeting of pSTAT5 in MPN-SC is efficacious in MPN patients remains unknown.

## 1. Introduction

Classical myeloproliferative neoplasms (MPN) are incurable stem cell disorders characterized by the abnormal expansion of myeloid cells in the bone marrow (BM), elevated blood counts, extramedullary myelopoiesis, and a genetic instability with enhanced risk to transform to secondary acute myeloid leukemia (sAML) [1,2,3,4,5,6]. In most patients, a mutation in the *calreticulin* (*CALR*) gene or the *Janus kinase 2* (*JAK2*) point mutation V617F is found [3,4,5,6]. MPN-related morbidity and mortality are emerging health problems in the Western world. Notably, improved diagnostics and therapy, together with an enhanced life expectancy, have led to an increasing prevalence of MPN. For patients with advanced MPN or sAML, the only curative approach is allogeneic hematopoietic stem cell transplantation [7,8,9]. However, this therapy can only be offered to a subset of patients. In all other cases, disease management is based on symptom control and the use of growth-inhibitory drugs, including interferon-alpha, anagrelide, hydroxyurea or ruxolitinib [10,11,12,13,14,15]. However, these drugs have little if any curative potential and in many cases resistance develops during therapy [10,11,12,13,14,15]. Therefore, current research is seeking new molecular targets and is attempting to develop new targeted drugs for patients with MPN.

Based on the classification of the World Health Organization (WHO), three types of classical MPN have been defined: polycythemia vera (PV), essential thrombocythemia (ET) and primary myelofibrosis (PMF) [3]. Each of these neoplasms exhibits unique clinical, histopathological and molecular features [1,2,3,4,6]. However, the three MPN entities share molecular and pathologic characteristics, and in many cases an overlap or transition from one into another type of MPN is seen. In most patients, mutations in the *JAK2*, *CALR* or *thrombopoietin receptor* (*MPL*) gene are found [16,17,18,19,20]. Independent of the disease variant, JAK2 activation leads to a cascade of downstream signaling molecules and pathways in neoplastic cells [21,22,23,24,25]. One of the key downstream signaling molecules is the ’signal transducer and activator of transcription-5’ (STAT5) protein [21,22,23,24,25,26,27].

Although STAT5 was initially characterized as a key transcription factor in various physiologic and pathologic processes, more recent data suggest that STAT5 also serves as a cytoplasmic signaling molecule that binds to and interacts with other signaling molecules in neoplastic cells to promote oncogenesis. Both the cytoplasmic and the nuclear fractions of STAT5 are considered to critically contribute to leukemogenesis in patients with myeloid neoplasms [28,29,30].

Similar to other myeloid neoplasms, MPN are considered to develop from transformed myeloid stem cells [31,32,33,34,35]. As only the neoplastic stem cells of an MPN (MPN-SC) can propagate the malignancy for unlimited time periods, they represent an important cellular target of therapy. However, little is known about the phenotype and target expression profiles of neoplastic stem cells in MPN. As in other myeloid neoplasms, MPN-SC are considered to reside within the CD34^+^/CD38^−^ population of the clone [32,33,35]. It has also been described that the immature CD34^+^ cells in MPN patients express *JAK2* V617F [35].

The aims of the present study were to examine MPN cells for expression of phosphorylated (p) STAT5, to study the cellular distribution of pSTAT5 and to analyze the effects of pSTAT5-targeting drugs on MPN cells. Our data show that pSTAT5 is expressed in CD34^+^/CD38^−^ MPN stem cells and serves as a potential therapeutic target in MPN.

## 2. Results

### 2.1. Primary MPN Cells Express Nuclear and Cytoplasmic pSTAT5

**Figure 1:**
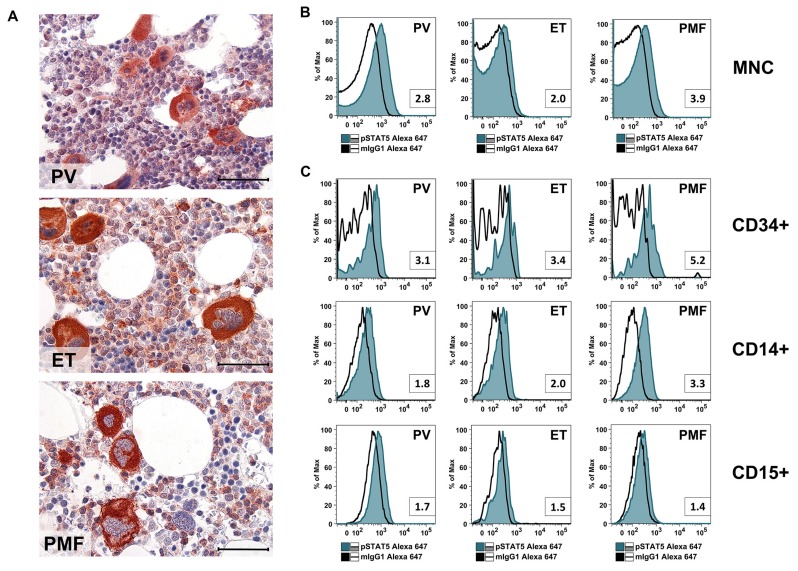
(**A**) Sections prepared from paraffin-embedded bone marrow (iliac crest) of patients with polycythemia vera (PV; patient #06), essential thrombocythemia (ET; patient #34) or primary myelofibrosis (PMF; patient #29) were stained with an anti-phosphorylated signal transducer and activator of transcription-5 (pSTAT5) antibody using immunohistochemistry. Examples of nuclear- and cytoplasmic staining are shown in Figure A1. Scale bar: 30 µm. Patient characteristics are shown in Table A1. (**B**,**C**) Bone marrow (BM) mononuclear cells (MNC) of patients with PV (patient #30), ET (patient #08) or PMF (patient #29) were stained with an anti-pSTAT5 Alexa-647 antibody. Intracellular expression levels of pSTAT5 were analyzed by flow cytometry in total MNC (**B**), or in cell subsets gated for CD34, CD14 or CD15 (**C**). The isotype-matched control antibody is also shown (open black histogram). Numbers in the small boxes represent the staining index defined as the ratio of the median fluorescence intensity (MFI) obtained with the anti-pSTAT5 antibody and MFI obtained with the isotype-matched control antibody (mIgG1).

As assessed by immunohistochemistry (IHC), primary MPN cells in the BM of patients with PV, ET and PMF expressed pSTAT5 in their nuclear and cytoplasmic compartment (Figure 1A and Table 1). The expression of pSTAT5 in normal BM cells (controls) was similar to that found in MPN BM sections examined by IHC. In all samples tested, megakaryocytes stained clearly positive for pSTAT5 (positive control), whereas erythroid cells stained negative for pSTAT5 (negative control). We were also able to confirm expression of cytoplasmic pSTAT5 in BM cells in patients with various MPN by multi-color flow cytometry (Figure 1B). In these experiments, all myeloid cells tested, including CD15^+^ granulomonocytic cells, CD14^+^ monocytes and CD34^+^ stem and progenitor cells, were found to stain positive for pSTAT5 (Figure 1C). pSTAT5 was identified in BM cells in all three categories of MPN, regardless of expression of *JAK2* V617F and without major differences in staining intensities (Figure 1B, Table 1).

### 2.2. Primary CD34^+^/CD38^−^ MPN-SC Express pSTAT5

**Figure 2:**
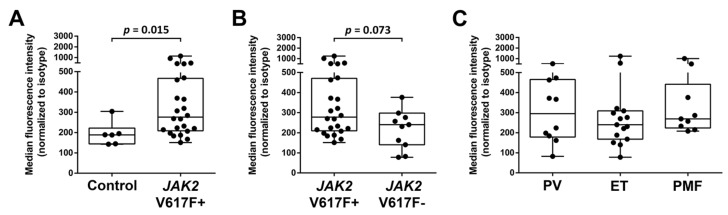
Bone marrow cells from patients with PV, ET or PMF were analyzed for intracellular expression of pSTAT5 in CD34^+^/CD38^−^/CD45^dim^ cells using an anti-pSTAT5 Alexa-647 antibody. (**A**) Expression of pSTAT5 in normal/reactive bone marrow (Control, *n* = 6) and bone marrow of MPN patients (MPN, *n* = 24). (**B**) Expression of pSTAT5 in CD34^+^/CD38^−^/CD45^dim^ bone marrow cells in *JAK2* V617F+ patients (V617F+, *n* = 24) and patients with wild type *JAK2*, a *CALR* mutation or an *MPL* mutation (V617F−, *n* = 10). (**C)** Expression of pSTAT5 in CD34^+^/CD38^−^/CD45^dim^ bone marrow cells in the three different MPN subgroups (PV, *n* = 10; ET, *n* = 15, PMF, *n* = 9). Boxes indicate the upper and lower quartiles, the median is defined by a horizontal line inside the boxes, and the whiskers show the highest and lowest values.

Expression of pSTAT5 in CD34^+^/CD38^−^ cells was examined in normal/reactive BM cells and in BM samples obtained from patients with MPN by multi-color flow cytometry. As visible in Figure 2, pSTAT5 was found to be expressed in normal CD34^+^/CD38^−^ stem cells as well as in CD34^+^/CD38^−^ MPN-SC. We also found that pSTAT5 levels were higher in *JAK2* V617F+ CD34^+^/CD38^−^ MPN-SC compared to normal stem cells (*p* = 0.015) (Figure 2A). In addition, we found that pSTAT5 is expressed at slightly higher levels in CD34^+^/CD38^−^ MPN-SC in *JAK2* V617F+ patients compared to *JAK2* V617F- patients, although the difference was not statistically significant (*p* = 0.073) (Figure 2B). However, no substantial differences in pSTAT5 expression in CD34^+^/CD38^−^ MPN cells were found when comparing various subsets of MPN (PV vs. ET vs. PMF) (Figure 2C).

### 2.3. Detection of pSTAT5 in HEL and SET-2 Cells

**Figure 3:**
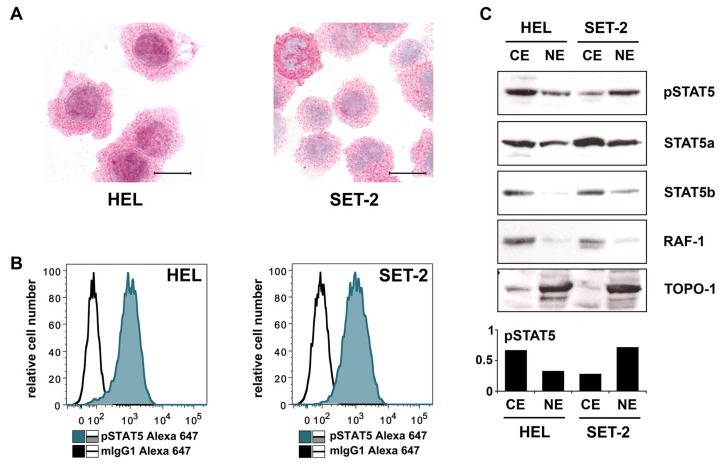
(**A**) Cytospin preparations of HEL cells and SET-2 cells were stained overnight with an anti-pSTAT5 antibody using immunocytochemistry. Scale bar: 20 µM. (**B**) HEL cells and SET-2 cells were stained with an anti-pSTAT5 Alexa-647 antibody for 30 min at room temperature and intracellular expression levels were analyzed by flow cytometry. (**C**) Nuclear (NE) and cytoplasmic (CE) fractions of HEL or SET-2 cells were analyzed for expression of phosphorylated STAT5 (pSTAT5), total STAT5a and total STAT5b by Western blotting. Antibodies against RAF-1 (cytoplasm) and TOPO-1 (nucleus) were used as fraction controls. The columns show the densitometry for pSTAT5. Uncropped blots are shown in Figure S1.

We next examined the *JAK2* V617F+ cell lines HEL and SET-2 by immunocytochemistry (ICC). Confirming previous studies [36,37], we found that both cell lines express pSTAT5. Interestingly, both cell lines expressed pSTAT5 in their nuclear and cytoplasmic compartments (Figure 3A). Intracellular expression of pSTAT5 in these cell lines was also confirmed by flow cytometry (Figure 3B). Preincubation of HEL and SET-2 cells with JAK2-targeting drugs (ruxolitinib [38,39], R763 [40], TG101348 [41,42], AZD1480 [43,44]) or STAT5-targeting drugs (piceatannol [45], pimozide [46,47], AC-3-019 [48], AC-4-130 [48,49]) resulted in reduced pSTAT5 staining (Figure A2). The characteristics of the STAT5- and JAK2-targeting drugs used in these experiments together with their main targets are depicted in Table A2. We also compared pSTAT5 levels in purified nuclear and cytoplasmic fractions of HEL and SET-2 cells by Western blotting. Total STAT5 (STAT5A and STAT5B) was expressed more abundantly in the cytoplasmic extracts of HEL and SET-2 cells than in nuclear extracts (Figure 3C). Moreover, pSTAT5 was found at higher levels in the cytoplasm of HEL cells compared to nuclear fractions. By contrast, in SET-2 cells, pSTAT5 was found to be expressed more abundantly in the nuclear fractions compared to cytoplasmic fractions (Figure 3C).

### 2.4. Effects of JAK2 V617F and CALR Mutants on Expression of Total STAT5 and pSTAT5 in Ba/F3 Cells

**Figure 4:**
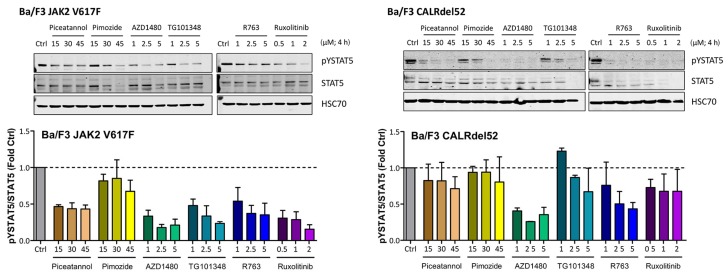
Ba/F3 cells modified to express JAK2 V617F or CALRdel52 were incubated with various JAK2 or STAT5 inhibitors (as indicated) for four hours at 37 °C. Thereafter, the expression of activated STAT5 (pSTAT5), total STAT5 (STAT5) and heat shock cognate 70 (HSC70), which was used as loading control, was analyzed using Western blotting. The results were quantified using densitometry and are expressed as the ratio of activated and total STAT5 normalized to the results obtained with control cells (Ctrl). Uncropped blots are shown in Figure S2.

To explore the mechanism of expression and activation of STAT5 in MPN cells, we expressed *JAK2* V617F and mutated *CALR* in Ba/F3 cells containing human *MPL* (Ba/F3-MPL). As expected, expression of JAK2 V617F was followed by an increase in pSTAT5 levels in these cells (Figure A3). Moreover, we were able to show that drugs targeting STAT5 (piceatannol, pimozide) or JAK2 (AZD1480, TG101348, R763, ruxolitinib) counteract mutant-induced overexpression of pSTAT5 in our Ba/F3-MPL cells (Figure 4).

### 2.5. Effects of Targeted Drugs on the Growth and Survival of HEL and SET-2 Cells

**Figure 5:**
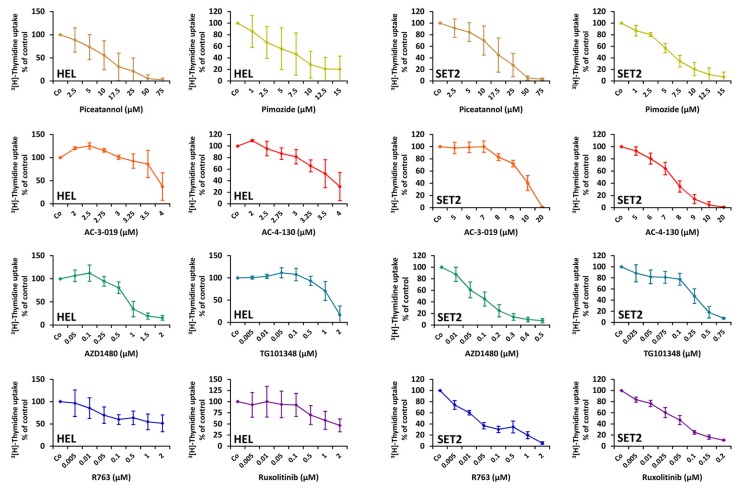
HEL and SET-2 cells were incubated with different concentrations of JAK2 or STAT5 targeting drugs for 48 h at 37 °C. Thereafter, ^3^[H]-thymidine was added for 16 h and its incorporation was analyzed using a beta counter. The diagrams show the mean ± standard deviation (SD) of at least three independent experiments performed in triplicate.

To confirm that pSTAT5 serves as a molecular target in MPN cells, we applied various JAK2-targeting drugs and STAT5-targeting drugs on HEL cells and SET-2 cells. As shown in Figure 5, the JAK2 blockers (ruxolitinib, R763, TG101348, AZD1480) and the STAT5 blockers (piceatannol, pimozide, AC-3-019, AC-4-130) were found to inhibit ^3^[H]-thymidine uptake and thus proliferation with varying potency. For several drugs, these data confirmed previous studies [50,51,52,53]. In our study, the rank order of potency for HEL cells was AZD1480 > TG101348 > ruxolitinib > R763 > AC-4-130 > AC-3-019 > pimozide > piceatannol. The rank order of potency for SET-2 cells was R763 > ruxolitinib > AZD1480 > TG101348 > pimozide > AC-4-130 > AC-3-019 > piceatannol. Next, we examined the effects of various targeted drugs on the survival of MPN cells. In these experiments, we found that the JAK2 and STAT5 blockers examined induce apoptosis in HEL cells and SET-2 cells (Figure A4). In a separate set of experiments, we applied ruxolitinib and AC-4-130 in combination in HEL and SET-2 cells. However, no clear additive or synergistic effects of this drug combination was observed (Data not shown [54]). We also examined the effects of the JAK2 and STAT5 inhibitors on proliferation of two pSTAT5-low/negative solid cancer cell lines, A2780 and A375. As shown in Table A3, these cells were in general less sensitive to these drugs compared to HEL cells or SET2 cells.

### 2.6. Effects of Targeted Drugs on Primary Human MPN Cells

**Figure 6:**
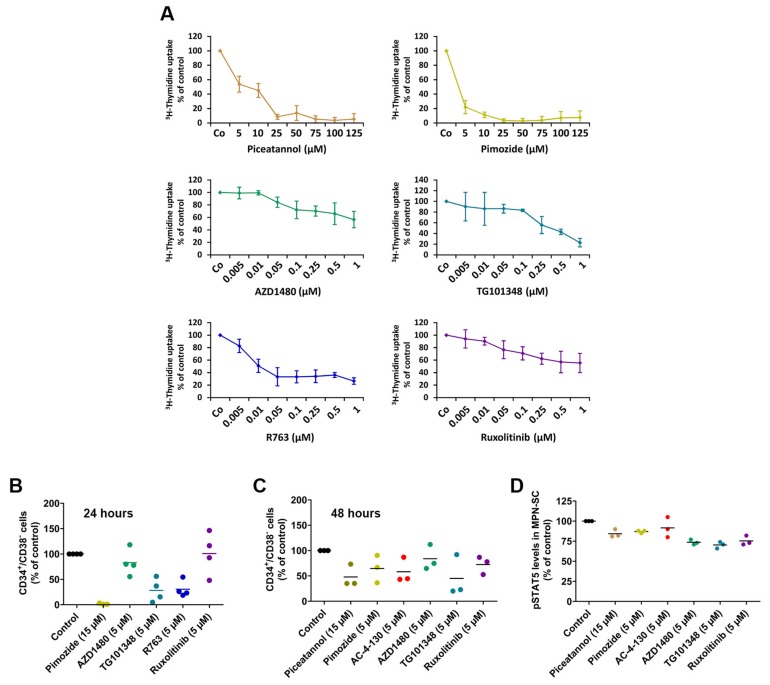
(**A**) Mononuclear cells isolated from the bone marrow of MPN patients were incubated with different concentrations of JAK2 or STAT5 targeting drugs for 48 h. Thereafter, ^3^[H]-thymidine was added for 16 h and its incorporation was analyzed using a beta counter. The diagrams show the mean ± SD of three independent experiments for JAK2-targeting drugs (patients #07, #20 and #23) or five independent experiments for STAT5-targeting drugs (patients #20, #23, #37, #35 and #39). (**B,C**) Mononuclear cells isolated from the bone marrow of MPN patients were incubated with different concentrations of JAK2 or STAT5 targeting drugs for 24 h (**B**) or 48 h (**C**). Then, the numbers of putative MPN stem cells (MPN-SC), defined as CD34^+^/CD38^−^/CD45^dim^, were assessed using counting beads. The graphs show MPN-SC as percent of control in four patients (#14, #29, #30 and #31) in (B) and in three patients (#31, #42 and #43) in (**C**). Horizontal lines indicate mean values. (**D**) pSTAT5 levels in CD34^+^/CD38^−^ MPN-SC determined by flow cytometry (same patients as in (C)) after an incubation with targeted drugs (as indicated) for 4 h. Values represent pSTAT5 levels relative to the control. The mean staining index is also shown (horizontal bars). Patient characteristics are shown in Table A1.

In a final step, we examined the effects of various JAK-2-targeting drugs and STAT5-targeting drugs on the growth and survival of primary mononuclear cells (MNC) obtained from the BM of patients with MPN. In these experiments, we found that the JAK-2 blockers and the STAT5 blockers used in this study are capable of inhibiting the proliferation of primary MPN cells (Figure 6A). Moreover, we found that these drugs decrease the relative numbers of primary CD34^+^/CD38^−^ MPN-SC in vitro (Figure 6B,C). Interestingly, ruxolitinib was found to decrease MPN-SC numbers after 48 h but not after 24 h. All drugs also decreased pSTAT5 levels in MPN-SC, although the downregulating effects of these drugs were rather weak (Figure 6D).

## 3. Discussion

Recent data suggest that STAT5 activation is a critical event triggering oncogenesis and growth of neoplastic cells in various hematologic malignancies [55,56]. It has also been described that leukemia-specific oncoproteins, such as BCR-ABL1, induce activation of STAT5 and thereby contribute to clonal expansion of neoplastic cells [57]. Several studies have shown that phosphorylated STAT5 (pSTAT5) is expressed in the cytoplasm and nuclei of neoplastic cells in patients with chronic myeloid leukemia (CML) and systemic mastocytosis (SM) [28,58]. Our study shows that neoplastic cells in PV and PMF also express nuclear and cytoplasmic pSTAT5 in a constitutive manner. In addition, we found that putative CD34^+^/CD38^−^ MPN-SC display pSTAT5. Finally, our data show that pharmacologic targeting of STAT5 reduces the growth of MPN cells and the numbers of MPN-SC. Together, these data suggest that the STAT5 pathway may contribute to oncogene-dependent growth of neoplastic cells in MPN. Whether inhibitors of pSTAT5, alone or in combination with other drugs, can exert clinically meaningful effects in MPN patients, remains unknown at present.

Recent data suggest that pSTAT5 is detectable in the cytoplasm of neoplastic cells in patients with AML, CML and SM, and that STAT5 acts as a pro-oncogenic driver in these myeloid neoplasms [28,29,30,58,59,60]. An interesting observation has been that the levels of cytoplasmic pSTAT5 often exceed the amounts of pSTAT5 found in the nuclear compartments in these cells [28,58]. In the current study, we found that pSTAT5 is located in both the cytoplasmic and nuclear fractions of primary MPN cells and in the MPN-related cell lines HEL and SET-2. In both cell lines, cytoplasmic and nuclear pSTAT5 were detected by ICC, flow cytometry and Western blotting (Figure 3). As assessed by Western blotting, the cytoplasmic and nuclear fractions of these cells displayed detectable levels of pSTAT5, and total STAT5 was found to be expressed more abundantly in the cytoplasmic fraction. Currently, it remains unknown whether nuclear pSTAT5 or cytoplasmic pSTAT5 plays a more important role in oncogenesis in MPN. Whereas nuclear pSTAT5 is considered to act as a pro-oncogenic transcription factors, cytoplasmic pSTAT5 may be involved in pro-oncogenic signaling involving the PI3 kinase pathway, similar to the situation in AML and SM [29,61].

MPN cells are considered to be organized in a ‘stem cell hierarchy’ similar to normal hematopoiesis [34]. In addition, MPN cells can undergo differentiation and terminal maturation in the same way as normal myeloid cells. As in normal hematopoiesis, only the most immature neoplastic stem cells (MPN-SC) have the capacity of self-renewal in MPN, a hypothesis that has major implications regarding drug therapy [62,63]. Notably, this model predicts that these cells can propagate the malignancy for unlimited time periods and that anti-neoplastic drugs have curative potential only when eliminating most or all of these neoplastic stem cells in a given neoplasm. So far, only little is known about the phenotype and function of MPN-SC [35,64]. Like in other myeloid neoplasms, these cells are considered to reside in a CD34^+^ fraction of the clone [31]. In the present study, we were able to show that pSTAT5 is not only expressed in more mature clonal MPN cells, but also in immature CD34^+^/CD38^−^ (putative) MPN-SC (Figure 1 and Figure 2). To the best of our knowledge, our study is the first to demonstrate that CD34^+^/CD38^−^ MPN-SC express pSTAT5. Regarding more mature clonal cells, monocytes, known to play a major role in MPN [65,66,67], were found to express high levels of pSTAT5, making these cells an additional potential target of STAT5 inhibition. As assessed by flow cytometry, we also found that the levels of pSTAT5 are higher in putative MPN-SC in patients with *JAK2* V617F^+^ MPN compared to patients with *JAK2* V617F^−^ MPN or normal CD34^+^/CD38^−^ stem cells. In contrast, no statistically significant difference was observed when comparing patients with *JAK2* V617F^−^ MPN or normal CD34^+^/CD38^−^ stem cells, although some patients with *JAK2* V617F^−^ MPN showed higher pSTAT5 levels than normal controls. Interestingly, when testing for pSTAT5 levels by IHC, no differences were found between the BM of MPN patients and normal controls, which seems to be in contrast to our flow cytometry results. However, our flow cytometry staining experiments were performed on stem cells, whereas IHC was performed on the bulk of MPN cells. Moreover, the IHC stain may be less capable of precisely quantifying differences in staining intensities compared to flow cytometry. This hypothesis is supported by the work of Teofili et al. [68], who did not detect differences in pSTAT5 levels between different subsets of MPN when using IHC in bulk cells. By contrast, using a flow cytometry approach, Abba et al. [69], were able to show differences in pSTAT5 levels when comparing CD34^+^ MPN cells with normal CD34^+^ cells.

In our study, no differences were seen when comparing pSTAT5 expression levels in CD34^+^/CD38^−^ MPN-SC among the three groups of MPN patients, and in each case, pSTAT5 was homogenously expressed in all MPN-SC in all patients. Collectively, these data suggest that neoplastic stem and progenitor cells in MPN patients express pSTAT5. As mentioned above, our results are also in line with the data published by Abba et al. [69], who showed that pSTAT5 levels are higher in CD34^+^ MPN cells compared to normal CD34^+^ cells. However, Abba et al. did not look into the more immature fraction of CD34^+^/CD38^−^ stem cells [69].

A number of previous and more recent studies have shown that STAT5 activation is a critical event triggering oncogenesis in MPN (stem) cells and that mutated forms of JAK2 and CALR can induce STAT5 activation [22,23,24,25]. In our study, we were able to show that putative SC in MPN express pSTAT5, but these cells only expressed pSTAT5 in excess over normal stem cells in patients with *JAK2*-mutated MPN. These data suggest that STAT5 may play a particular role in MPN-SC downstream of JAK2. However, more studies are required to define the exact role that STAT5 activation plays in the immature stem cell compartment in MPN.

**Figure A1:**
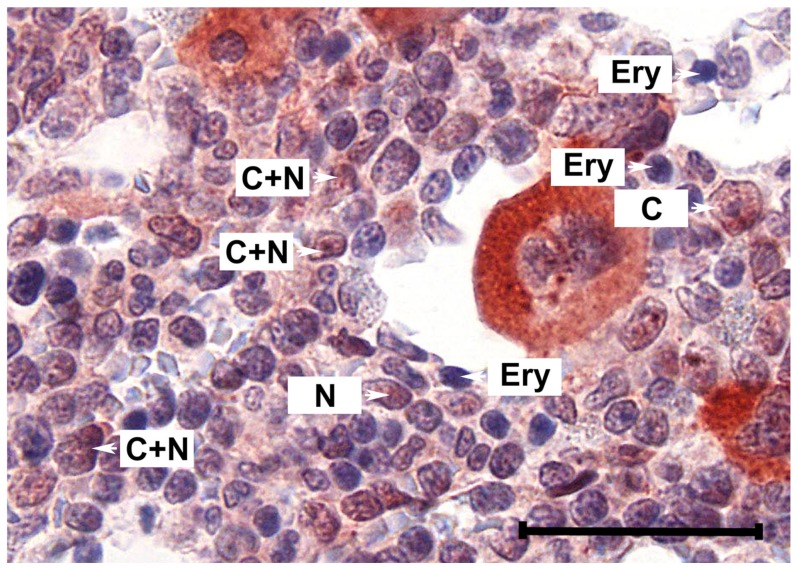
The picture shows an enlarged part of the picture of patient #06 (PV) from Figure 1A. The arrows point at examples of cytoplasmic ‘C’, nuclear ‘N’ and cytoplasmic/nuclear ‘C+N’ staining. ‘Ery’ marks examples of erythroid cells that were used as internal negative controls. Scale bar: 30 µm.

**Figure A2:**
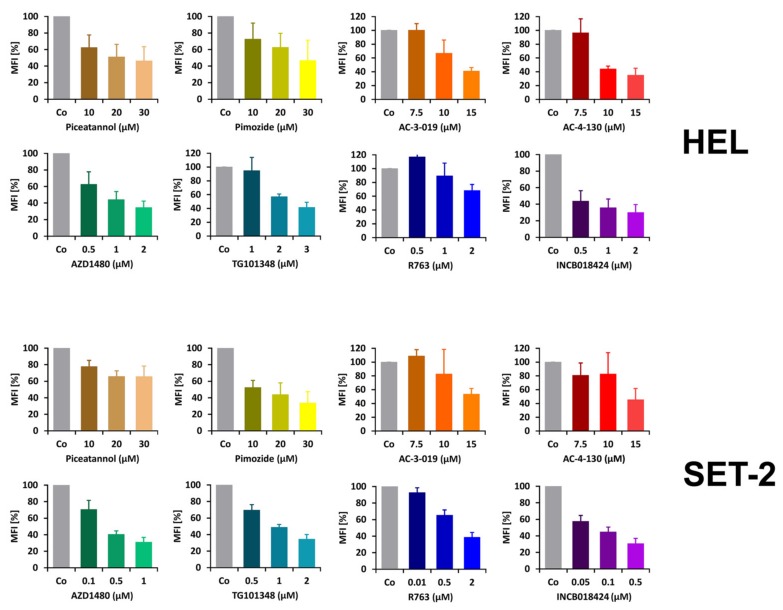
HEL and SET-2 cells were incubated with various concentrations of JAK2-targeting drugs (ruxolitinib, R763, TG101348, AZD1480) or STAT5-targeting drugs (piceatannol, pimozide, AC-3-019, AC-4-130) for 4 h. Thereafter, expression of pSTAT5 was analyzed by multicolor flow cytometry. The levels of pSTAT5 are depicted as median fluorescence index (MFI) values relative to the untreated control (Co).

**Figure A3:**
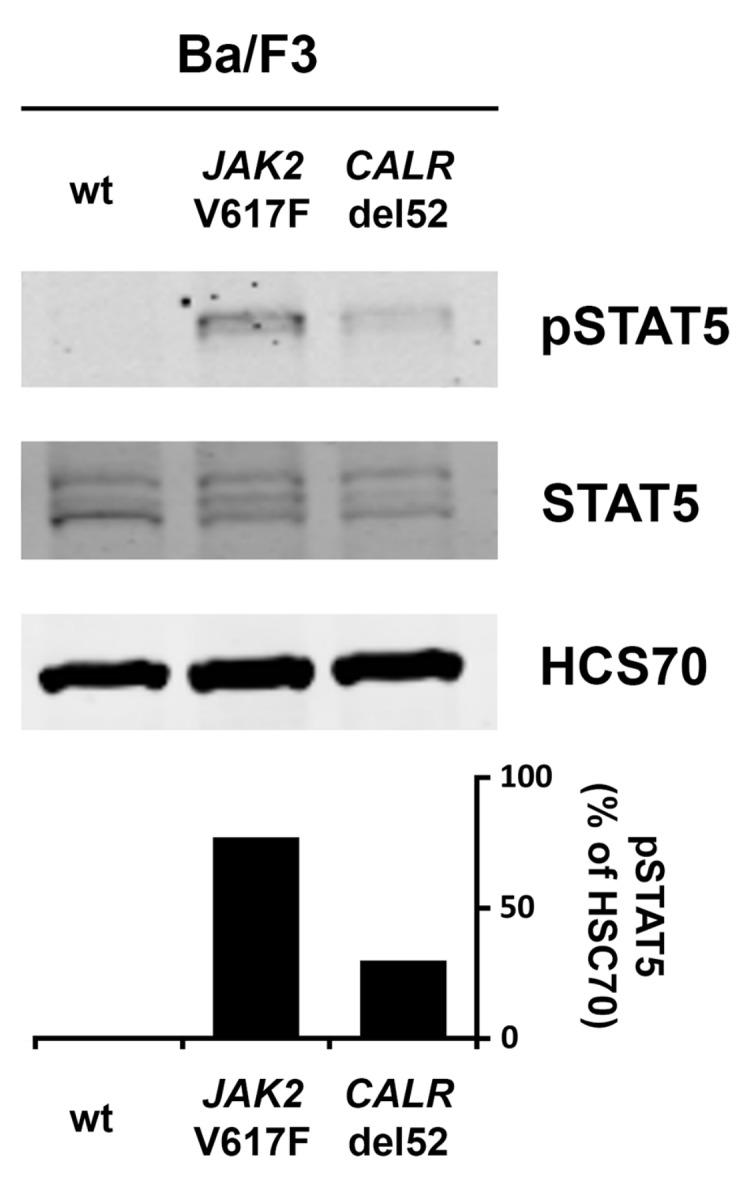
Wild type (wt) Ba/F3 cells or Ba/F3 cells transfected with either *JAK2* V617F or *CALRdel52* were incubated overnight in medium without IL-3. After, cell lysates were subjected to Western blotting using antibodies against pSTAT5, STAT5 and HSC70. The columns show the levels of pSTAT5 in percent of HSC70 as analyzed by densitometry. Uncropped blots are shown in Figure S3.

**Figure A4:**
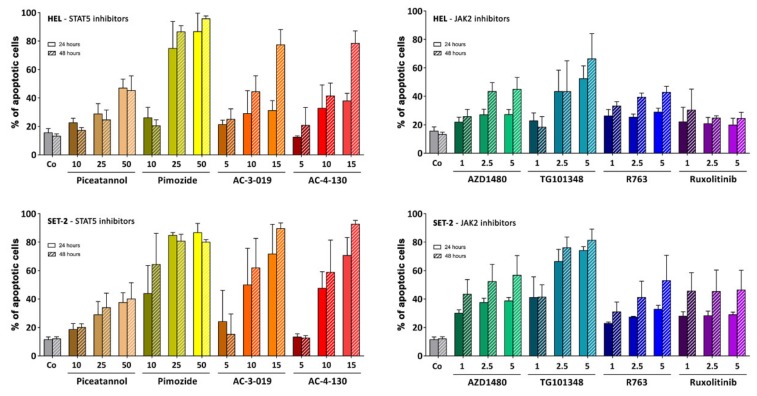
HEL and SET-2 cells (as indicated) were incubated with different concentrations of JAK2 or STAT5 targeting drugs for 24 h or 48 h at 37 °C. Thereafter, apoptosis induction was assessed using Annexin V/propidium iodide (PI) staining. The columns show the sum of cells positive for Annexin V-only, PI-only or Annexin V/PI for each condition and are depicted as mean ± SD of at least three independent experiments.

Inhibitors of JAK2 and STAT5 were found to suppress expression of pSTAT5 in HEL and SET-2 cells (Figure A1). For several of these drugs, our data confirm the available literature [50,51,52,53]. An interesting observation was that whereas the JAK2-targeting drugs blocked STAT5 activation and MPN cell growth at relatively low concentrations, much higher concentrations of the STAT5 blockers were required to counteract proliferation in MPN cells (Figure 5). These observations suggest that targeting of STAT5 may be an interesting approach to block oncogenic signaling in MPN cells directly, but more potent and specific STAT5 inhibitors need to be developed to better inhibit MPN cell growth. Indeed, multiple attempts have been made recently to develop selective and potent STAT5 inhibitors. In the present study, we examined the effects of two such novel STAT5 inhibitors, AC-3-019 and AC-4-130 [49]. Similar to piceatannol and pimozide, these selective STAT5-SH2-domain-targeting drugs produced growth inhibition and apoptosis, albeit at relatively high concentrations (Figure 5 and Figure A4). We also observed the effects of these inhibitors and of the other STAT5 or JAK2 blockers on MPN-SC. However, the effects on MPN-SC were weak, so these drugs are not expected to be able to eradicate the disease. One explanation for this result may be that other downstream signaling molecules and pathways are also involved in triggering oncogenesis and neoplastic stem cell growth in MPN. Therefore, we believe that more specific and more potent STAT5 blockers may be a reasonable approach to target MPN cells, and additional drugs or drug combinations, as proposed by Bar-Natan et al. [53] and others, may be required to elicit optimal anti-neoplastic or even curative effects.

## 4. Materials and Methods

### 4.1. Patients

Thirty-four patients with MPN, including 10 with PV, 15 with ET and 9 with PMF were examined for levels of pSTAT5 in MPN-SC. MPN variants were diagnosed according to WHO criteria [3]. Patient characteristics are shown in Table A1. Routine staging included physical examination, blood counts, morphologic examination of cells on BM smears, BM histology and immunohistochemistry, and analysis of BM and peripheral blood cells for expression of *JAK2* V617F, *CALR* and *MPL* mutations. Expression of *JAK2* V617F was determined by two PCR assays: the qualitative Ipsogena MutaScreen assay and the quantitative MutaQuant assay. Both assays were applied according to the recommendations of the manufacturer (Qiagen, Venlo, Netherlands). In *JAK2* V617F-negative patients, MPN cells were screened for *CALR* mutations using fluorescence- based PCR, followed by Sanger sequencing of codons 351–404 of *CALR* as described [16]. When no *JAK2* or *CALR* mutations were detected, the patients were analyzed for *MPL* mutations using an allelic discrimination assay as described [20]. In 15 patients (PV, *n* = 4; ET, *n* = 5; PMF, *n* = 6), BM mononuclear cells (MNC) were enriched using Ficoll. These MNC were used to assess drug effects on proliferation and/or survival. In addition, BM cells obtained from eight donors without MPN (control BM samples) were analyzed (Table A4). All investigations were approved by the local ethics committee of the Medical University of Vienna (ethic code: 224/2006; 1184/2014; 1063/2018). Informed consent was obtained from all patients.

### 4.2. Antibodies (Ab) and Other Reagents

**Figure A5:**
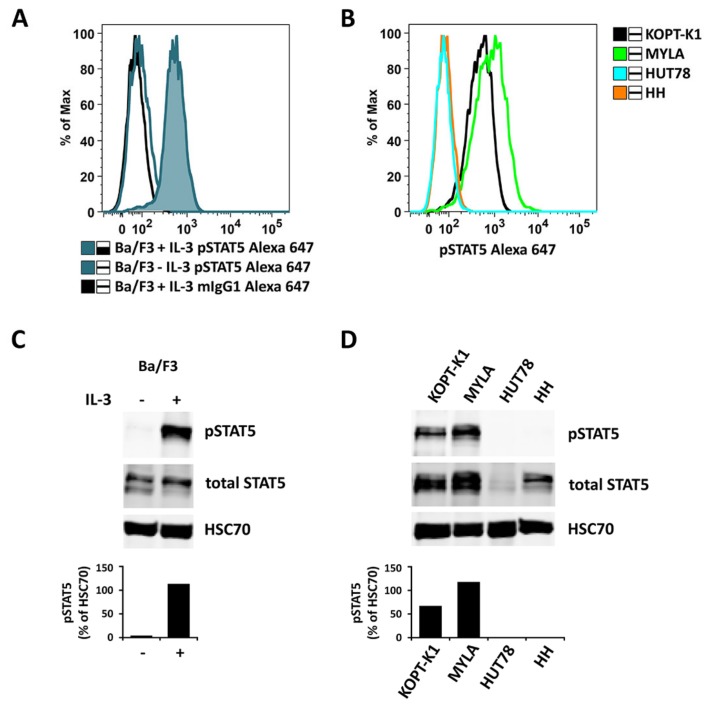
Ba/F3 cells were kept in medium without IL-3 overnight and split in the morning. One half was incubated with 10 ng/mL IL-3 for 40 min (Ba/F3 + IL-3), while the other half was kept in medium without IL-3 (Ba/F3 − IL-3). Thereafter, cells were analyzed for expression of pSTAT5 either by flow cytometry (**A**) or Western blotting (**C**). In (**A**), an Alexa-647-labeled anti-pSTAT5 antibody was used (with IL-3: blue histogram; without IL-3: open blue histogram). mIgG1 Alexa-647 was used as an isotype control (open black histogram) in Ba/F3 + IL-3 cells (**A**). Human lymphoma cell lines with detectable pSTAT5 levels (KOPT-K1 and MYLA) or no detectable pSTAT5 (HUT78 and HH) were also analyzed for pSTAT5 expression using flow cytometry (**B**) and Western blotting (**D**). In Western blot experiments, HSC70 served as a loading control. The columns show the levels of pSTAT5 in percent of HSC70 as analyzed by densitometry. Uncropped blots are shown in Figure S4.

The anti-pSTAT5 alpha (Tyr694) polyclonal Ab was purchased from Invitrogen (order number 71-6900; Carlsbad, CA, USA), anti-pSTAT5 (Y694) Alexa Fluor^®^ 647 monoclonal Ab (mAb) 47 (order number: 612599) and an isotype-matched control antibody (mIgG1-Alexa Fluor^®^ 647, order number: 557783) from BD Biosciences Pharmingen (San Jose, CA, USA), piceatannol (order number: P0453) and pimozide (order number: P1793) from Merck (Darmstadt, Germany), and the JAK2 blockers AZD1480 (order number: CT-A1480), TG101348 (order number: CT-TG101) and ruxolitinib (order number: CT-INCB) from ChemieTek (Indianapolis, IN, USA). The specificity of pSTAT5 (Y694) Alexa Fluor^®^ 647 was confirmed by flow cytometry experiments performed with human cell lines with detectable pSTAT5 (KOPT-K1 and MYLA) or no detectable pSTAT5 (HUT78 and HH). In addition, we employed Ba/F3 cells with IL-3-inducible expression of pSTAT5. In these experiments, the specificity of the antibody was confirmed (Figure A5a,b). pSTAT5 expression in the cell lines tested was confirmed by Western Blotting (Figure A5c,d). Roswell Park Memorial Institute (RPMI) 1640 medium (order number: 10-041-CVR) was purchased from Corning (Manassas, VA, USA) and fetal calf serum (FCS; order number: 10270-106) from Gibco (Karlsbad, CA, USA). The JAK2 and Aurora kinase-targeting drug R763 was kindly provided by Yasumichi Hitoshi (Rigel Pharmaceuticals, San Francisco, CA, USA). The STAT5 inhibitors AC-3-019 and AC-4-130 were produced as described [48]. Stock solutions of drugs were prepared by dissolving in dimethyl sulfoxide (DMSO) (order number: D2650; Merck).

### 4.3. Cell Lines

A375 were purchased from LGC Standards (Wesel Germany); HEL and SET-2 cell lines were purchased from Deutsche Sammlung von Mikroorganismen und Zellkulturen (Braunschweig, Germany); KOPT-K1 cells were kindly provided by Takaomi Sanda (Cancer Science Institute of Singapore, Singapore); HH, HUT78 and MYLA were kindly provided by Marco Herling (University Hospital Cologne, Cologne, Germany). A2780 cells were purchased from Sigma (St. Louis, MO, USA). All cell lines were cultured in RPMI 1640 medium supplemented with 10% FCS.

### 4.4. Immunohistochemistry and Immunocytochemistry

Immunohistochemistry was performed on paraffin-embedded, formalin-fixed BM biopsy specimens using the indirect immunoperoxidase staining technique following established protocols [28]. Endogenous peroxidase was blocked by methanol/H_2_O_2_ and heat-induced epitope retrieval was performed (96 °C, 20 min, pH 9). A polyclonal anti-pSTAT5 antibody was applied at 1:100 for 20 h at 4 °C. Biotinylated goat anti-rabbit IgG (Vector Laboratories, Burlingham, CA, USA, order number: BA-1000) was applied as secondary antibody for 30 min at room temperature. Then, slides were exposed to an avidin/biotinylated peroxidase complex (Vectastain ABC Kit from Vector, order number: PK-6100) for 30 min. The chromogen 3-amino-9-ethylcarabzole (AEC) was then used. Finally, slides were counterstained in Mayer’s hematoxylin (Morphisto, Frankfurt am Main, Germany, order number: 10231.01000). In each sample, the specificity of the anti-pSTAT5 stain was controlled by analyzing the internal negative control and positive control. In fact, erythroid cells always stained negative and megakaryocytes always stained positive as reported previously [28]. Immunocytochemistry was performed using HEL and SET-2 cells as described [28]. Cells were spun on cytospin slides and fixed with acetone for 8 min. Slides were pretreated in citrate buffer (pH 6.0) at 95 °C for 20 min and incubated with a polyclonal anti-pSTAT5 antibody (Invitrogen, Carlsbad, CA, USA order number: 71-6900) diluted 1:100, overnight at 4 °C. Slides were incubated with biotinylated goat anti-rabbit IgG (Biocare Medical, Walnut Creek, CA, USA) for 30 min at room temperature (RT) and then with streptavidin AP label (Biocare Medical, Walnut Creek, CA, USA) for 30 min. Neofuchsin (Nichirei, Tokyo, Japan) was used as the chromogen. All slides were counterstained in Mayer´s hematoxylin.

### 4.5. Flow Cytometry

**Figure A6:**
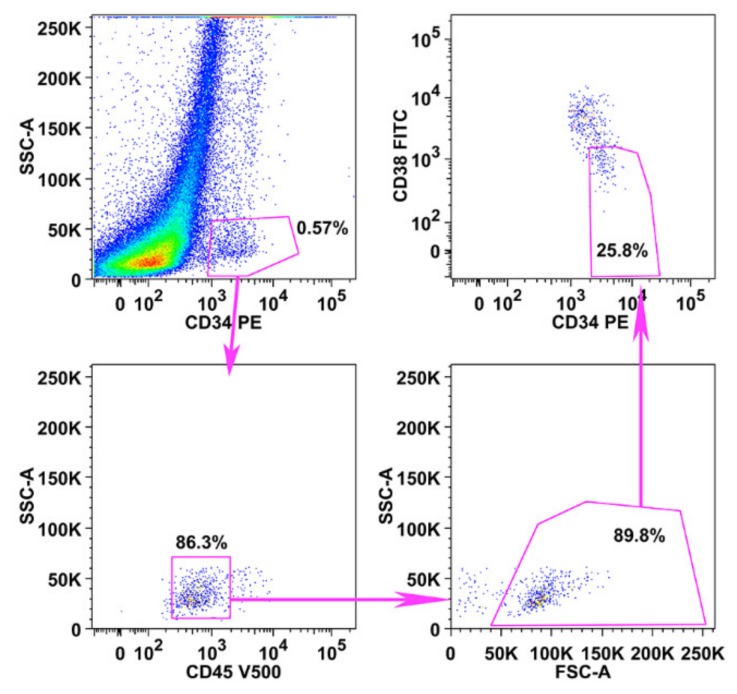
To identify immature CD34^+^/CD38^−^ stem cells (SC), we performed a sequential gating algorithm, indicated by pink arrows using FlowJo software. After excluding CD45-negative cells, immature cells were identified by their expression of CD34 (upper left panel) and were then gated as CD45 dim-positive blast cells (lower left panel). Thereafter, viable cells were selected by their light-scatter properties (lower right panel) and were then gated according to the lack (pink gate) of CD38 (upper right panel). The example shows bone marrow cells from patient #23.

Expression of cell surface antigens on primary neoplastic stem cells was analyzed by multicolor flow cytometry using antibodies against CD34, CD38 and CD45 as described [70]. Putative stem cells were defined as CD34^+^/CD38^−^/CD45^dim^. The gating strategy is shown in Figure A6. For assessing the absolute numbers of stem cells by flow cytometry, Absolute Counting Beads (Thermo Fisher Scientific, Waltham, MA, USA, order number: C36950) were used according to the instructions by the manufacturer. For the flow cytometric detection of cytoplasmic pSTAT5, cells were first stained for cell surface antigens and then fixed in formaldehyde (2%). Cells were subsequently permeabilized by exposure to 50% methanol (−20 °C, 10 min), washed in phosphate-buffered saline containing 0.1% bovine serum albumin (order number: A4503; Merck) and stained with the Alexa 647-conjugated anti-pSTAT5 mAb 47 pY694 or an isotype-matched control antibody for 30 min at RT. Cells were then washed and analyzed on a FACSCanto (BD Biosciences). Staining reactions were expressed as median fluorescence intensity (MFI). pSTAT5 expression levels are shown as staining index (SI) defined as MFI produced by anti-pSTAT5 antibody:MFI of the isotype-matched control antibody.

### 4.6. Western Blot Analysis of Expression of pSTAT5 in Ba/F3 Cells

To confirm the selective effects of JAK2 or STAT5 targeting drugs, Western blot experiments were performed using the pSTAT5 antibody Tyr694 on Ba/F3 and Ba/F3-MPL cells engineered to express *JAK2* V617F and *CALR*del52, respectively. Ba/F3 cells were generated as described [71,72]. Cells were incubated with drugs targeting JAK2 (AZD1480, TG101348, ruxolitinib, R763; 1–5 µM) or STAT5 (piceatannol, pimozide; 10–45 µM) for 4 h. Then, pSTAT5 expression was analyzed by Western blotting essentially as described [49]. Nitrocellulose membranes (0.45 µm Amersham Protran; order number: 10600002; GE Healthcare, Buckinghamshire, UK) were incubated with the following antibodies at the dilution indicated: polyclonal rabbit anti-phospho-STAT5 (Y694) antibody (order number: 71-6900; 1:1000; Invitrogen, Camarillo, CA, USA), monoclonal mouse anti-STAT5 antibody 89/Stat5 (order number: 610191; 1:1000; BD Biosciences), mouse anti-HSC70 monoclonal antibody B-6 (order number: SC-7298; 1:10,000; Santa Cruz, St. Louis, MO, USA), IRDye^®^ 680RD goat anti-rabbit IgG (order number: 925-68071; 1:10000; LI-COR, Lincoln, NE, USA), and IRDye^®^ 800CW goat anti-mouse IgG (order number: 925-32210; 1:10000; LI-COR). pSTAT5 levels were quantified by densitometry and expressed as the pSTAT5/loading control ratio normalized to control (untreated) cells.

### 4.7. Isolation of the Cytoplasmic and Nuclear Fractions of HEL and SET-2 Cells

HEL and SET-2 cells were lysed in hypotonic buffer (20 mM HEPES, 10 mM KCl, 1 mM ethylenediaminetetraacetic acid, 0.2% NP40, 10% glycerol, 5 μg/mL aprotinin, 5 μg/mL leupeptin, 1 mM phenylmethylsulfonide fluoril, and 1 mM Na_2_VO_4_). Cell lysates were centrifuged (5 min, 800 × *g*) to separate cytoplasmic and nuclear fractions [61]. Supernatants (cytoplasmic fraction) were frozen at −70 °C. Pelleted nuclei were resuspended in hypertonic buffer (hypotonic buffer plus 350 mM NaCl) and protein extracts were prepared by agitation (30 min, 4 °C). After debris was removed by centrifugation, nuclear extracts were frozen at −70 °C. Expression of pSTAT5 was determined by Western blotting and quantified by densitometry as reported [61]. Fractionation of subcellular compartments was controlled by applying anti-RAF-1 (cytoplasmic) and anti-topoisomerase-1 (nuclear) antibodies in parallel. All antibodies were from Santa Cruz.

### 4.8. Evaluation of Drug Effects on the Growth and Survival of MPN Cells

To further determine the functional role of STAT5 in MPN cells, we applied several targeting drugs: piceatannol, pimozide, AZD1480, TG101348, R763, ruxolitinib, AC-3-019 and AC-4-130 (Table A2). Primary human MPN cells (ET, *n* = 2; PMF, *n* = 3; PV, *n* = 2), HEL cells and SET-2 cells were incubated with increasing drug concentrations at 37 °C for 48 h. Thereafter, ^3^[H]-thymidine was added, and its uptake was analyzed after 16 h using a beta-counter. For evaluation of apoptosis, HEL and SET-2 cells were incubated in control medium or in various drug concentrations for 24 h and 48 h at 37 °C. The percentage of apoptotic cells was quantified using Annexin V/Propidium Iodide staining as described [73]. In a subset of patients, we examined drug effects on putative CD34^+^/CD38^−^ MPN-SC.

## 5. Conclusions

STAT5 is a critical molecule in MPN cells that acts downstream of oncogenic JAK2 V617F and mutant CALR. We found that pSTAT5 is expressed abundantly in the nuclear and cytoplasmic compartment of MPN cells and that pSTAT5 is not only present in more mature clonal cells, but also in putative CD34^+^/CD38^−^ MPN-SC. Moreover, we show that STAT5 expression correlates with survival of MPN cells and that drugs targeting STAT5 can block growth and survival of these cells. Since MPN-SC display STAT5, and STAT5 is downstream of both JAK2 V617F and mutant CALR, targeting of STAT5 may be a promising approach to treat MPN.

## Figures and Tables

**Table 1:** Immunohistochemical detection of pSTAT5 in bone marrow cells of MPN patients and controls.

Diagnosis	PV	PV	PV	PMF	PMF	PMF	ET	ET	ET	nBM	nBM	nBM	nBM
Patient #	18	06	30	20	29	16	34	02	23	46	48	50	51
Megakaryocytes	++	++	++	+	++	++	++	++	+	++	++	++	++
Myeloid prog.	+	+	+	+	+	+	+	+/−	+/−	+	+(+)	+	+
Neutrophil gran.	+	+	+/−	+	+	+	+	−	+/−	+/−	+	−	+/−
Eosinophil gran.	−	−	−	−	−	−	−	−	n.a.	−	−	n.a.	n.a.
Erythroid prog.	−	−	−	−	−	−	−	−	−	−	−	−	−

Score: ++, strong expression in most cells; +, clear expression in the majority of cases; +/-, expressed in subsets of cells or only weakly expressed; −, no expression (below detection limit). Abbreviations: CM, cutaneous mastocytosis; ET, essential thrombocythemia; gran., granulocytes; MPN, myeloproliferative neoplasm; n.a., not analyzed (no cells found by microscopy); nBM, normal bone marrow; NHL, Non-Hodgkin lymphoma; PMF, primary myelofibrosis; prog., progenitors; PV, polycythemia vera.

## Supplementary Materials

The following are available online at https://www.mdpi.com/2072-6694/12/4/1021/s1, Figure S1: Uncropped Blots of Figure 3C, Figure S2: Uncropped Blots of Figure 4, Figure S3: Uncropped Blots of Figure A3, Figure S4: Uncropped Blots of Figure A5.

## Appendix A

**Table A1:** Patient characteristics.

#	Sex	Age at Sampling	Diagnosis	Mutated *JAK2*, *CALR* or *MPL*	Mutation	pSTAT5 FACS	pSTAT5 IHC	Proliferation	SC Assay	pSTAT5 Regulation
01	f	44	PMF	*JAK2*	V617F	yes	-	-	-	-
02	f	61	ET	none	--	yes	yes	-	-	-
03	f	53	ET	none	--	yes	-	-	-	-
04	f	71	ET	*JAK2*	V617F	yes	-	-	-	-
05	f	76	PV	*JAK2*	V617F	yes	-	-	-	-
06	f	76	PV	*JAK2*	V617F	yes	yes	-	-	-
07	f	53	PV	none	--	yes	-	yes	-	-
08	f	61	ET	*CALR*	ins5: c.1154_1155insTTGTC, p.Lys385Asnfs*47	yes	-	-	-	-
09	f	50	PMF	*JAK2*	V617F	yes	-	-	-	-
10	f	64	PMF	*JAK2*	V617F	yes	-	-	-	-
11	f	74	ET	*JAK2*	V617F	yes	-	-	-	-
12	f	52	PMF	*CALR*	ins5: c.1154_1155insTTGTC, p.Lys385Asnfs*47	yes	-	-	-	-
13	f	50	ET	*MPL*	W515L	yes	-	-	-	-
14	f	71	ET	*JAK2*	V617F	yes	-	-	yes	-
15	m	67	PV	none	--	yes	-	-	-	-
16	m	70	PMF	*CALR*	del52: c.1099_1150del, p. Leu367Thrfs*46	yes	yes	-	-	-
17	f	37	ET	none	--	yes	-	-	-	-
18	f	66	PV	*JAK2*	V617F	yes	yes	-	-	-
19	m	74	PV	*JAK2*	V617F	yes	-	-	-	-
20	m	53	PMF	*JAK2*	V617F	yes	yes	yes	-	-
21	m	71	PV	*JAK2*	V617F	yes	-	-	-	-
22	m	51	PMF	*JAK2*	V617F	yes	-	-	-	-
23	m	43	ET	*JAK2*	V617F	yes	yes	yes	-	-
24	m	59	ET	*CALR*	del52: c.1099_1150del, p. Leu367Thrfs*46	yes	-	-	-	-
25	f	84	ET	none	--	yes	-	-	-	-
26	m	37	ET	*JAK2*	V617F	yes	-	-	-	-
27	f	45	PMF	*JAK2*	V617F	yes	-	-	yes	-
28	f	31	ET	*JAK2*	V617F	yes	-	-	-	-
29	f	85	PMF	*JAK2*	V617F	yes	yes	-	yes	-
30	f	74	PV	*JAK2*	V617F	yes	yes	-	yes	-
31	f	80	PV	*JAK2*	V617F	yes	-	-	yes	yes
32	f	72	ET	*JAK2*	V617F	yes	-	-	-	-
33	m	49	PV	*JAK2*	V617F	yes	-	-	-	-
34	m	64	ET	*JAK2*	V617F	yes	yes	-	-	-
35	f	71	PMF	*JAK2*	V617F	-	-	yes	-	-
36	f	68	ET	*JAK2*	V617F	-	-	yes	-	-
37	f	48	PMF	*JAK2*	V617F	-	-	yes	-	-
38	f	34	PV	*JAK2*	V617F	-	-	yes	-	-
39	m	88	PMF	*CALR*	del52: c.1099_1150del, p. Leu367Thrfs*46	-	-	-	yes	-
40	f	42	ET	*JAK2*	V617F	-	-	-	yes	-
41	f	38	ET	*JAK2*	V617F	-	-	-	yes	-
42	f	75	PV	*JAK2*	V617F	-	-	-	yes	yes
43	f	93	PMF	*JAK2*	V617F	-	-	-	yes	yes

*CALR*, *calreticulin*; ET, essential thrombocythemia; *JAK2*, *Janus kinase 2; MPL, MPL proto-oncogene, thrombopoietin receptor*; MPN, myeloproliferative neoplasm; PMF, primary myelofibrosis; Proliferation, effects of drugs on proliferation of MNC isolated from MPN patients; pSTAT5 FACS, sample analyzed for expression of pSTAT5 in CD34^+^/CD38^−^ putative stem cells; pSTAT5 IHC, sample analyzed by immunohistochemistry for expression of pSTAT5 in different bone marrow cells; pSTAT5 regulation, effects of JAK2 or STAT5 targeting drugs on pSTAT5 levels in CD34^+^/CD38^−^/CD45^dim^ cells; SC assay, effects of JAK2 or STAT5 targeting drugs on relative numbers of CD34^+^/CD38^−^/CD45^dim^ cells.

**Table A2:** Specifications of inhibitors used in this study.

Name [Ref]	Known Targets	Result of Target Inhibition (Drug Action)	Clinical Application	C_max_ in µM (at Dose/Day)	Concentration Range (µM) *	Supplier **
R763 [40]	JAK2, aurora kinase A, aurora kinase B, FLT3	G2/M phase arrest, endoreduplication	Phase 1 trial (human): 11.4–85.3 mg/day	0.09 (85.3 mg)	0.005–5	Rigel Pharmaceuticals
TG101348 [41,42]	JAK2, JAK1, JAK3, FLT3, RET, TYK2	proliferation inhibition, apoptosis induction	Phase 1 trial (human): 100–600 mg/day	2.8 (500 mg)	0.005–7.5	ChemieTek
AZD1480 [43,44]	JAK2, JAK1, JAK3, TYK2, FGFR3, STAT3	proliferation inhibition, apoptosis induction	Phase 1 trial (human): 5–80 mg/day	1.7 (30 mg)	0.005–7.5	ChemieTek
Ruxolitinib [38,39]	JAK2, JAK1, JAK3	proliferation inhibition, apoptosis induction	approved (human): PMF, PV (EU, USA)	2.3 (50 mg)	1–20	ChemieTek
Pimozide [46,47]	STAT5, D_2_ dopamine receptor, 5-HT_7_ receptor, Ca^2+^ channels	proliferation inhibition, apoptosis induction	approved (human): Tourette’s Syndrome (EU, USA)	0.007 (2 mg)	0.5–25	Merck
Piceatannol [45]	STAT5, SYK, LCK	proliferation inhibition, apoptosis induction, histamine release blockade	n.a.	8.1 (80.7 mg/kg)	1–75	Merck
AC-3-019 [48]	STAT5	proliferation inhibition, apoptosis induction	n.a.	n.a.	0.05–15	P. Gunning
AC-4-130 [48,49]	STAT5	proliferation inhibition, apoptosis induction	n.a.	n.a.	0.25–15	P. Gunning

C_max_, peak plasma concentration of a drug after administration; FGFR3, fibroblast growth factor receptor 3; FLT3, fms-like tyrosine kinase 3; JAK, janus kinase; LCK, leukocyte C-terminal SRC kinase; LYN, LCK/YES novel tyrosine kinase; n.a., not applicable; PMF, primary myelofibrosis; PV, polycythemia vera; Ref, Reference; STAT, signal transducer and activator of transcription; SYK, spleen tyrosine kinase; TYK2, tyrosine kinase 2; * Range of concentrations used in this study. ** ChemieTek (Indianapolis, IN, USA); LC Laboratories (Woburn, MA, USA); Merck, (Darmstadt, Germany); Rigel Pharmaceuticals (San Francisco, CA, USA); Patrick Gunning (Department of Chemistry, University of Toronto, Canada).

**Table A3:** IC_50_ concentrations of JAK2- and STAT5 targeting drugs in pSTAT5 high and low cell lines.

IC_50_ Obtained with Drugs in Various Cell Lines (SI pSTAT5)				
Inhibitor	HEL (13.4)	SET-2 (8.6)	A375 (1.93)	A2780 (1.97)
Piceatannol	10–17.5	10–17.5	>75	17.5–25
Pimozide	5–7.5	5–7.5	12.5–15	12.5–15
AC-3-019	3.5–4	9–10	10–12.5	12.5–15
AC-4-130	3.5–4	7–8	>15	>15
AZD1480	0.5–1	0.05–0.1	>2	0.5–1
TG1010348	1–2	0.1–0.25	>2	1–2
Ruxolitinib	1–2	0.0025–0.05	>2	>2

IC_50_ concentration: half maximal inhibitory concentration; SI: Staining Index (MFI produced by the anti-pSTAT5 antibody:MFI of the isotype-matched control antibody).

**Table A4:** Characteristics of control bone marrow donors.

#	Sex	Age at Sampling	Diagnosis	pSTAT5 FACS	pSTAT5 IHC
44	f	33	NHL	yes	-
45	m	37	nBM	yes	-
46	m	40	NHL	yes	yes
47	m	24	B-ALL (CR)	yes	-
48	f	47	CM	yes	yes
49	m	26	NHL	yes	-
50	f	33	NHL	-	yes
51	m	61	NHL	-	yes

B-ALL (CR), b-cell acute lymphoblastic leukemia in complete remission; CM, cutaneous mastocytosis; nBM, normal bone marrow; NHL, non-Hodgkin lymphoma; PMF, primary myelofibrosis; pSTAT5 FACS, sample analyzed for expression of pSTAT5 in CD34^+^/CD38^−^ putative stem cells; pSTAT5 IHC, sample analyzed by immunohistochemistry for expression of pSTAT5 in different bone marrow cells; PV, polycythemia vera.
