# Supplementary material for: STAT5 is Expressed in CD34+/CD38− Stem Cells and Serves as a Potential Molecular Target in Ph-Negative Myeloproliferative Neoplasms
Source: Cancers (Basel). 2020 Apr 21;12(4):1021. doi: 10.3390/cancers12041021 (PMC7225958; doi:10.3390/cancers12041021)
Supplement: Supplementary file 1 [file cancers-12-01021-s001.pdf]

Supplementary Materials: STAT5 is Expressed in CD34+/CD38− Stem Cells and Serves as a Potential Molecular Target in Ph-Negative Myeloproliferative Neoplasms

Emir Hadzijusufovic, Alexandra Keller, Daniela Berger , Georg Greiner, Bettina Wingelhofer, Nadine Witzeneder, Daniel Ivanov, Emmanuel Pecnard, Harini Nivarthi, Florian K. M. Schur, Yüksel Filik, Christoph Kornauth, Heidi A. Neubauer, Leonhard Müllauer, Gary Tin, Jisung Park, Elvin D. de Araujo, Patrick T. Gunning, Gregor Hoermann, Fabrice Gouilleux, Robert Kralovics, Richard Moriggl and Peter Valent

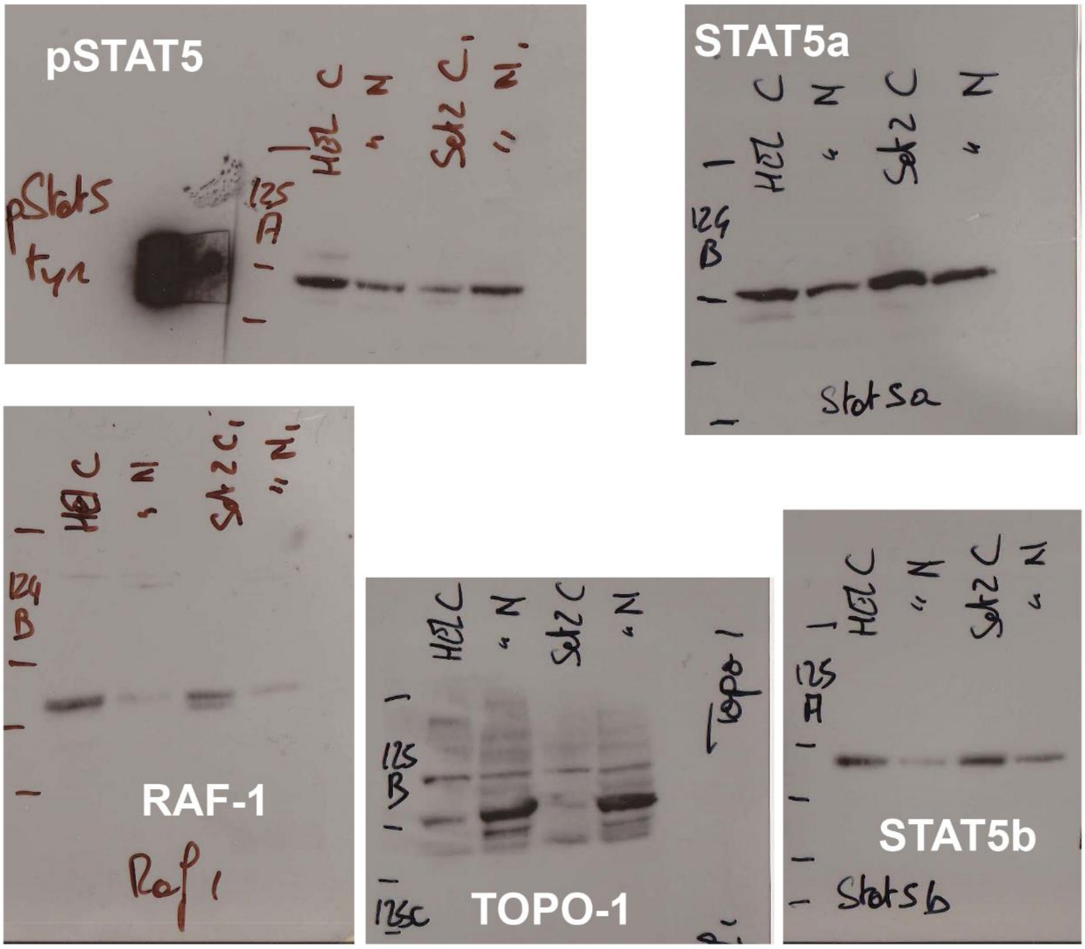

Figure S1. Uncropped Blots of Figure 3C.

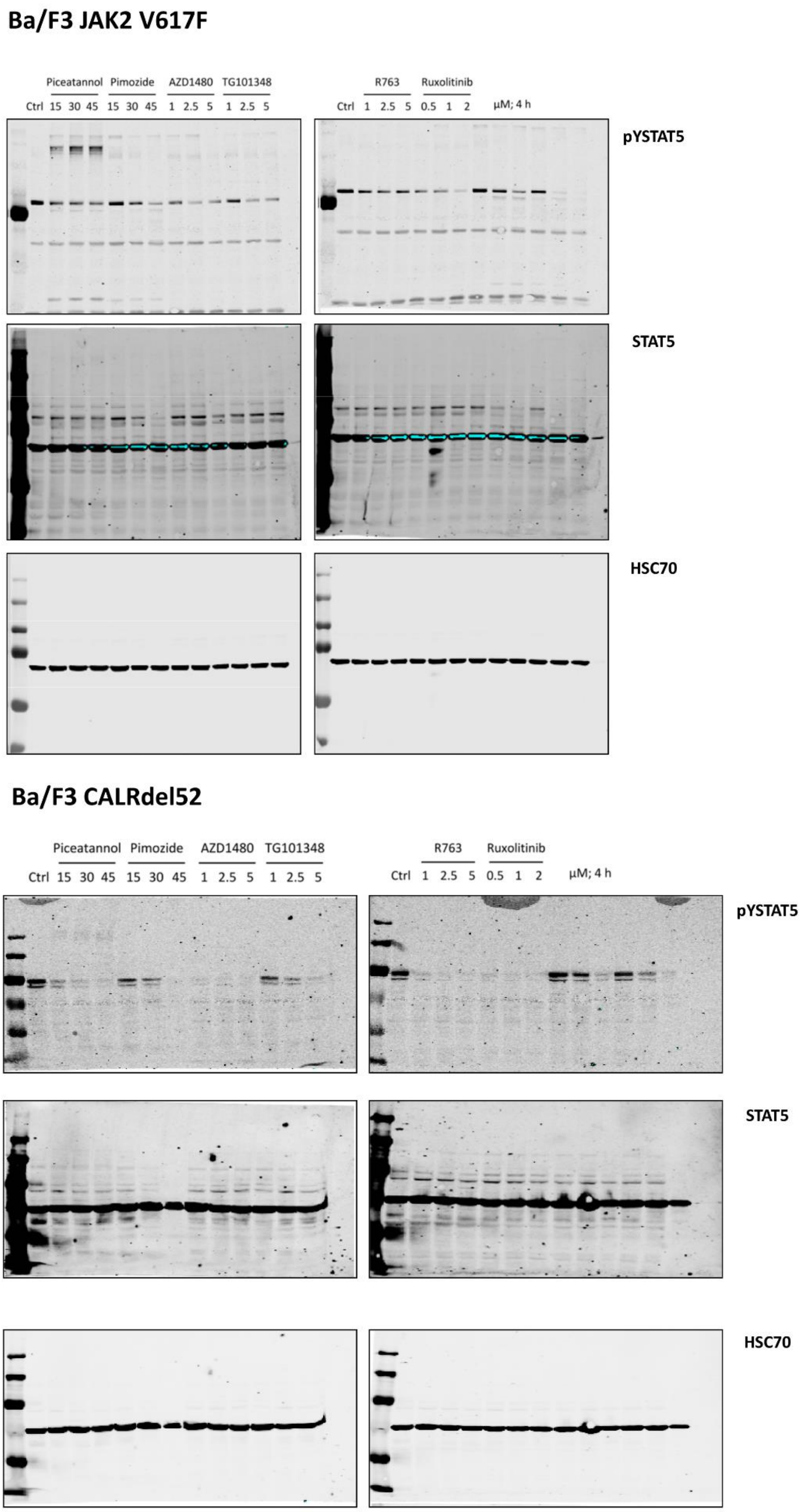

Figure S2. Uncropped Blots of Figure 4.

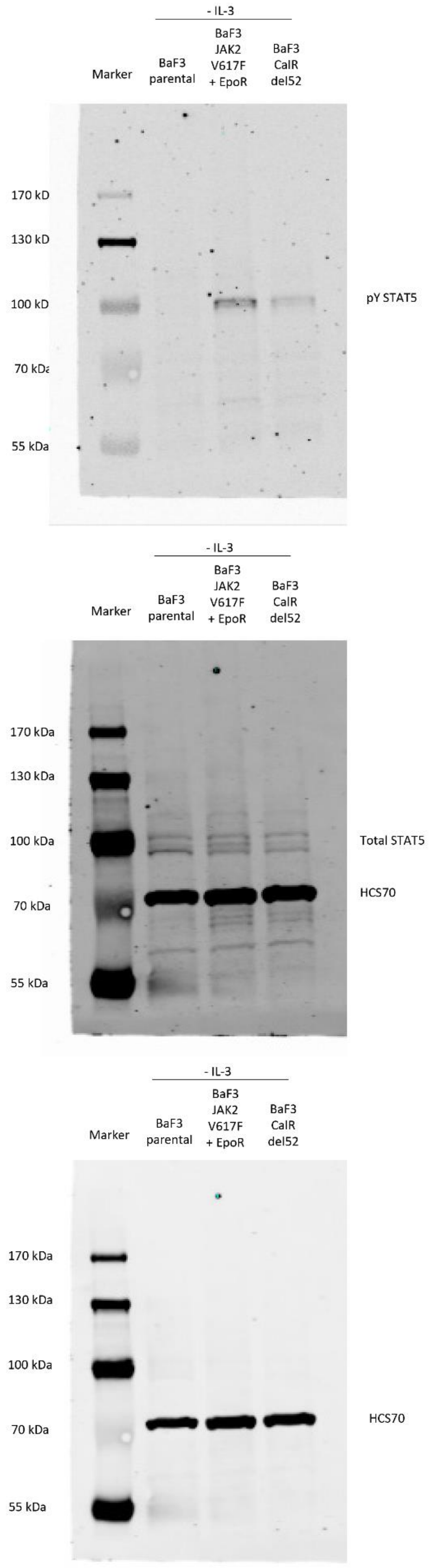

Figure S3. Uncropped Blots of Figure A3.

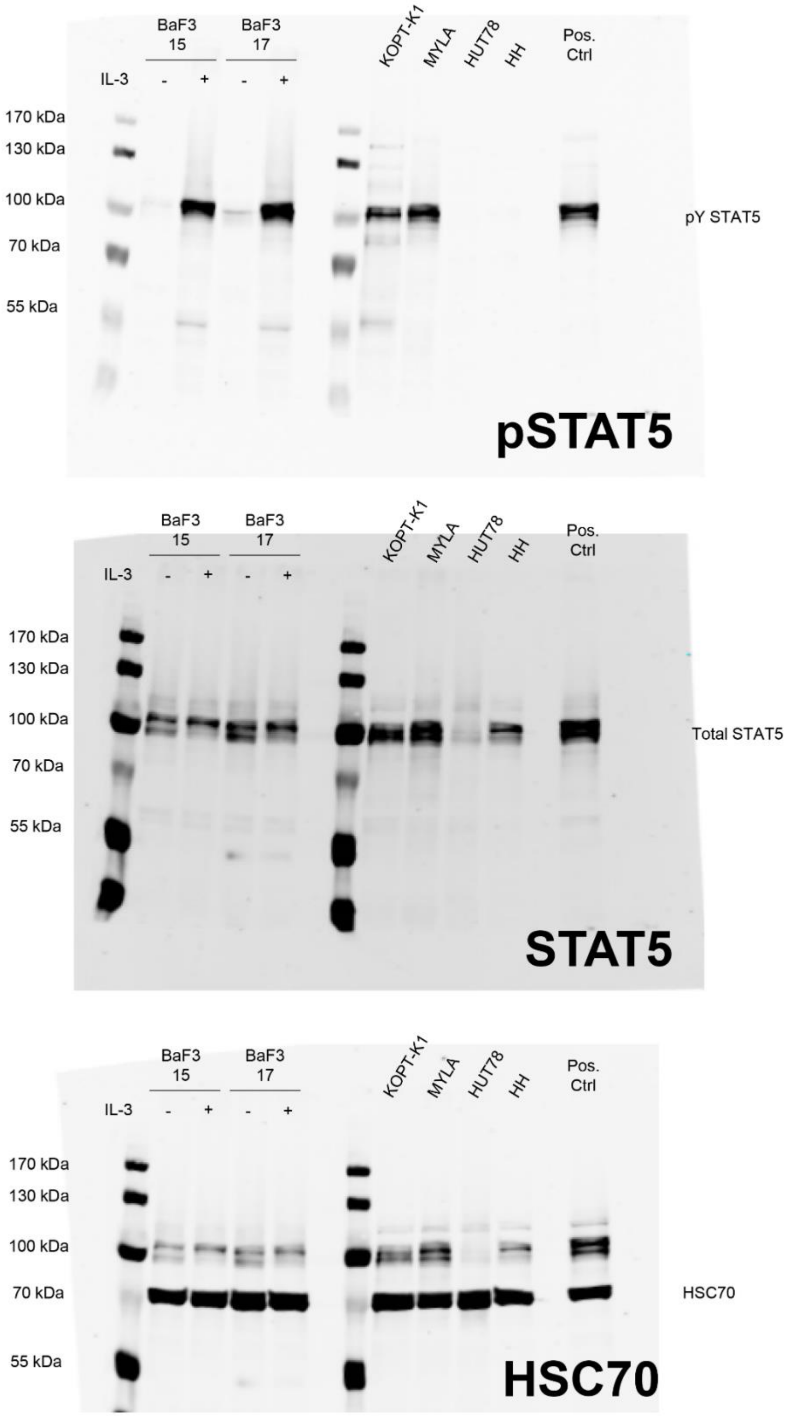

Figure S4. Uncropped Blots of Figure A5.

© 2020 by the authors. Licensee MDPI, Basel, Switzerland. This article is an open access article distributed under the terms and conditions of the Creative Commons Attribution (CC BY) license (http://creativecommons.org/licenses/by/4.0/).
